# Green synthesis of silver NPs using aqueous extract of *Artemisia scoparia* for hydrogenation of aromatic nitro compounds and their biological activity

**DOI:** 10.3389/fmicb.2025.1584066

**Published:** 2025-04-25

**Authors:** Prashant Bhati, Richa Tiwari, S. L. Kothari, Khushi Ray, Narendra Pal Lamba, Yogesh Kumar, Reem Binsuwaidan, Mohd Saeed, Safia Obaidur, Sonia Chahar Srivastava, Kumar Sambhav, Manmohan Singh Chauhan

**Affiliations:** ^1^Amity University Rajasthan, Jaipur, India; ^2^Department of Chemistry, Shivaji College, University of Delhi, New Delhi, India; ^3^Department of Pharmaceutical Sciences, College of Pharmacy, Princess Nourah bint Abdulrahman University, Riyadh, Saudi Arabia; ^4^Department of Biology, College of Science, University of Hail, Hail, Saudi Arabia; ^5^Department of Clinical Laboratory Sciences, College of Applied Medical Sciences, King Khalid University, Abha, Saudi Arabia; ^6^S.S. Jain Subodh PG College (Autonomous), Jaipur, India

**Keywords:** *Artemisia scoparia*, Silver nanoparticles (AgNPs), phytochemical screening, nanoparticle characterization, antibacterial activity, catalytic reduction

## Abstract

The green synthesis of silver nanoparticles (AgNPs) using *Artemisia scoparia* (*A. scoparia*), a plant abundant in bioactive compounds, provides an eco-friendly, cost-effective, and sustainable method for nanoparticle synthesis. Silver ions were successfully reduced using an aqueous extract of *A. scoparia*, resulting in AgNPs with a characteristic UV–visible absorption peak at 421 nm. Comprehensive characterization using FTIR, TEM, SEM, and DLS confirmed their stability, uniform morphology, and functional groups. The antibacterial activity of the synthesized AgNPs was evaluated against *E. faecalis* and *P. aeruginosa*, and their catalytic activity was assessed by reducing aromatic nitro compounds. *A. scoparia*-derived AgNPs demonstrated potent antibacterial activity, effectively inhibiting the growth of *E. faecalis* and *P. aeruginosa*, indicating potential for addressing antibiotic-resistant bacteria. Additionally, these AgNPs exhibited remarkable catalytic efficiency in reducing aromatic nitro compounds, showcasing their potential as eco-friendly catalysts. This dual functionality highlights their significant role in sustainable nanotechnology and environmental remediation efforts.

## 1 Introduction

Nanotechnology has become a transformative force in science and technology, offering solutions to some of the most pressing challenges in medicine, environmental remediation, and material science (Singh et al., 2024). Silver nanoparticles (AgNPs) are notable among various nanomaterials due to their remarkable characteristics, such as a high surface area-to-volume ratio, adjustable optical and electronic properties, and strong biological activities (Iwuji et al., 2024). AgNPs are widely recognized for their broad-spectrum antimicrobial and catalytic properties. These make them invaluable in diverse applications, ranging from antimicrobial coatings and drug delivery systems to environmental detoxification and renewable energy (More et al., 2023; Singh et al., 2015). Despite their versatility, conventional synthesis methods for AgNPs are often associated with significant drawbacks, including hazardous chemicals, high energy demands, and the generation of toxic byproducts. These limitations underscore the urgent need for environmentally sustainable approaches to nanoparticle production (Nie et al., 2023).

Green synthesis has emerged as an eco-friendly and sustainable alternative, utilizing biological entities such as plants, bacteria, fungi, and algae to facilitate nanoparticle synthesis (Bhardwaj et al., 2020). Among these, plant-based synthesis has garnered particular attention due to its simplicity, cost-effectiveness, and the rich diversity of bioactive metabolites in plant extracts (Gupta et al., 2023). For instance, plants such as *Azadirachta indica, Ocimum sanctum*, and *Artemisia scoparia* have been extensively studied for their effectiveness in synthesizing silver nanoparticles (AgNPs), owing to their abundant bioactive compounds (Alharbi et al., 2022). Plant-derived bioactive compounds, including phenols, flavonoids, terpenoids, and alkaloids, act as natural reducing, capping, and stabilizing agents, eliminating the need for external chemical additives (Mutha et al., 2021; Dias et al., 2021). This not only minimizes environmental impact but also enhances the biocompatibility and functionality of the synthesized nanoparticles. Green synthesis minimizes the use of hazardous chemicals, the potential toxicity of AgNPs on human health and the environment remains a critical concern. To ensure their safe application, future research should focus on evaluating the biocompatibility of these nanoparticles through cytotoxicity and genotoxicity assessments (Kirubakaran et al., 2025). Additionally, investigating the environmental fate of AgNPs, including their stability, potential accumulation in ecosystems, and effective disposal methods, is crucial for ensuring their sustainable use. Optimizing AgNP concentration for biomedical and environmental applications can further minimize toxicity risks while maintaining functional efficacy (Nie et al., 2023).

While several plants have been explored for green synthesis, existing methods often suffer from limitations such as low nanoparticle stability, inconsistent particle size distribution, or reduced antimicrobial efficiency. Furthermore, many reported methods require lengthy preparation steps or lack scalability for industrial applications (Ying et al., 2022). In this context, *Artemisia scoparia (A. scoparia)*, a medicinal plant known for its pharmacological potential, emerges as an ideal candidate for green nanoparticle synthesis. A member of the Asteraceae family, *A. scoparia* is renowned for its diverse bioactive profile, comprising phenols, flavonoids, sesquiterpenes, and essential oils (Ding et al., 2021). These compounds exhibit intrinsic antimicrobial, antioxidant, and anti-inflammatory properties, making the plant a valuable resource for nanoparticle synthesis (Parham et al., 2020; Li et al., 2024). Moreover, *A. scoparia's* unique phytochemical composition is hypothesized to improve nanoparticle stability, enhance antimicrobial efficacy, and provide better control over particle size distribution, addressing key issues observed in existing plant-based synthesis methods (Ding et al., 2021).

This study focuses on the green synthesis of AgNPs using an aqueous extract of *A. scoparia* as a natural reducing and stabilizing agent. Comprehensive characterization of the synthesized nanoparticles was performed using a range of advanced analytical techniques, including UV-vis spectroscopy, Dynamic Light Scattering (DLS), Zeta potential analysis, Fourier Transform Infrared (FTIR) spectroscopy, Scanning Electron Microscopy (SEM), and Transmission Electron Microscopy (TEM) (Vladár and Hodoroaba, 2020; Kumari et al., 2023). In parallel, the phytochemical composition of *A. scoparia* extract was elucidated using confirmatory tests identifying the key bioactive constituents responsible for nanoparticle synthesis and stabilization (Zhang et al., 2024; Ding et al., 2021; Kuerban et al., 2024).

The unique bioactive profile of *A. scoparia* ensures improved nanoparticle stability compared to conventional plant-based methods, while its synergistic combination of bioactive compounds enhances the antibacterial efficacy of the resulting AgNPs. This eco-friendly method also eliminates the need for toxic reducing agents, ensuring minimal environmental impact and offering a cost-effective solution for sustainable nanoparticle production (Fahim et al., 2024).

The biological and chemical activities of the synthesized AgNPs were also evaluated to explore their potential applications. The AgNPs demonstrated significant antibacterial properties, making them promising candidates for combating microbial infections (Rengarajan et al., 2024; Nie et al., 2023). The catalytic reduction of nitro compounds, a key environmental detoxification process, showed the remarkable chemical efficiency of the nanoparticles (Sassykova et al., 2019).

By integrating green synthesis, advanced characterization, and functional evaluation, this study not only underscores the potential of *A. scoparia* in the eco-friendly synthesis of AgNPs but also highlights the versatility of these nanoparticles in addressing biomedical and environmental challenges. This research contributes to the growing body of knowledge on plant-mediated nanoparticle synthesis, paving the way for sustainable and multifunctional nanomaterials in the future.

## 2 Experimental protocol and resources

### 2.1 Chemicals

All Analytical-grade reagents such as silver nitrate (AgNO_3_, ≥99.0%), 1-bromo-4-nitrobenzene, 4-nitroaniline, 4-bromo-2-fluoro-1-nitrobenzene, 4-nitrophenol, sodium borohydride (98%), were acquired from Merck (Germany and Mumbai, India), Acros Organics, and Scharlau (Barcelona, Spain), respectively. All solutions were prepared in deionized water, with glassware meticulously cleaned using nitric acid, rinsed in distilled and deionized water and oven-dried to prevent electrolyte interference (Ghojavand et al., 2020).

### 2.2 Plant sample collection and processing techniques

*A. scoparia* is an herbaceous plant characterized by its finely dissected leaves and aromatic scent. The plant typically exhibits yellowish-green foliage and produces small, inconspicuous flowers (Boakye et al., 2017). The classification of the plant is as follows:

Family: Asteraceae.

Subfamily: Asteroideae.

Genus: Artemisia.

Species: Scoparia.

Botanical name: *A. scoparia*.

Common name: Redstem Wormwood.

**Figure 1:**
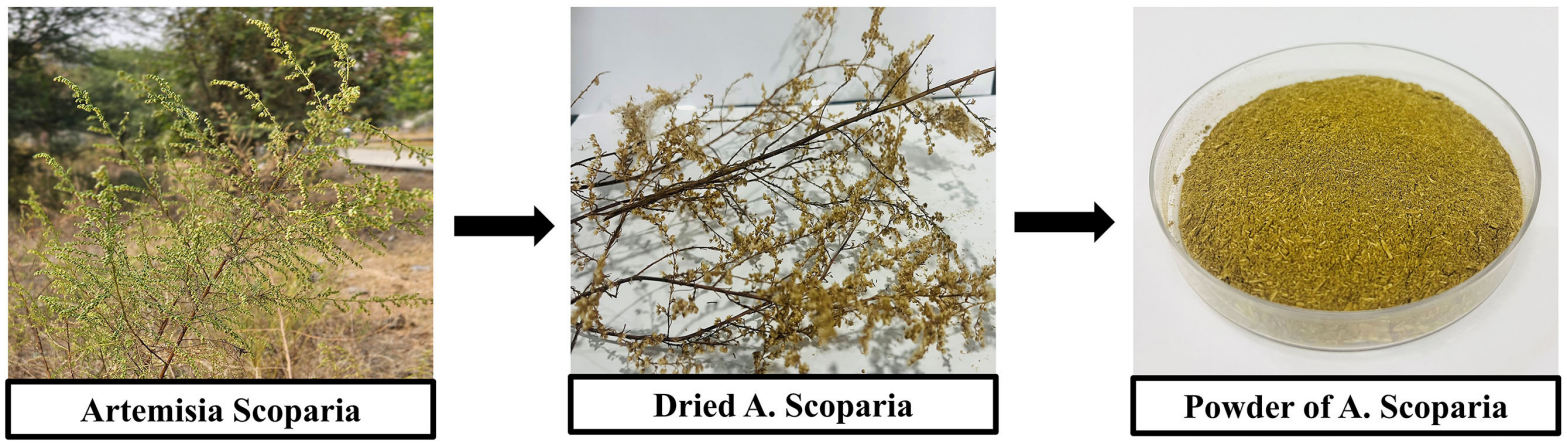
This figure shows the processing steps of *A. scoparia* for the preparation of its powder. The first image shows the fresh plant of *A. scoparia* in its natural habitat followed by the dried plant material in the second image. The final image displays the powdered form of *A. scoparia*, which is obtained after drying and grinding the plant material.

Freshly collected leaves and stems, from Amity University Rajasthan in Jaipur and thoroughly washed with distilled water to remove contaminants. The cleaned leaves and stems were dried in an oven at 60°C. For extract preparation, 10 g of the dried leaves and stems were ground into a fine powder (Figure 1) and mixed with 100 mL of deionized water in a 250 mL beaker. The mixture was boiled for 30 min and cooled to room temperature. The extract was filtered through Whatman No. 1 filter paper (pore size 25 μm), and centrifuged at 5,000 rpm for 15 min, the supernatant was filtered and further refined with 0.4 μm syringe filters. The final aqueous extract was stored in a clean bottle at 4°C for future synthesis applications.

### 2.3 Phytochemicals screening

#### 2.3.1 Confirmatory tests

Phytochemical screening is an essential qualitative test in herbal medicine and natural product research. It identifies key bioactive compounds, such as Phenols, Tannins, Flavonoids, Saponins, and Carbohydrates that play a vital role in plants' medicinal effects (Labulo et al., 2022).

#### 2.3.2 Test for phenols and tannins

**Ferric Chloride Test:** Take 1–2 mL of the plant extract in a test tube. Add 2–3 drops of 5% ferric chloride (FeCl_3_) solution to the extract. Mix gently and observe the color change.

#### 2.3.3 Test for flavonoids

**Alkaline Test:** Take 1–2 mL of the plant extract in a test tube. Add a few drops of 10% sodium hydroxide (NaOH) solution. Observe the color change. Add a few drops of dilute hydrochloric acid (HCl) to the mixture and observe again.

#### 2.3.4 Test for saponins

**Foam Test:** Take 1–2 mL of the plant extract in a test tube. Add 2–3 mL of distilled water. Shake the solution vigorously for about 1 min.

#### 2.3.5 Test for carbohydrates

**Molisch Test:** Take 2 ml of the plant extract in a test tube, add 2–3 drops of Molisch's reagent (a-naphthol), and then add concentrated sulfuric acid carefully along the side of the test tube.

### 2.4 Green synthesis of silver nanoparticles

**Figure 2:**
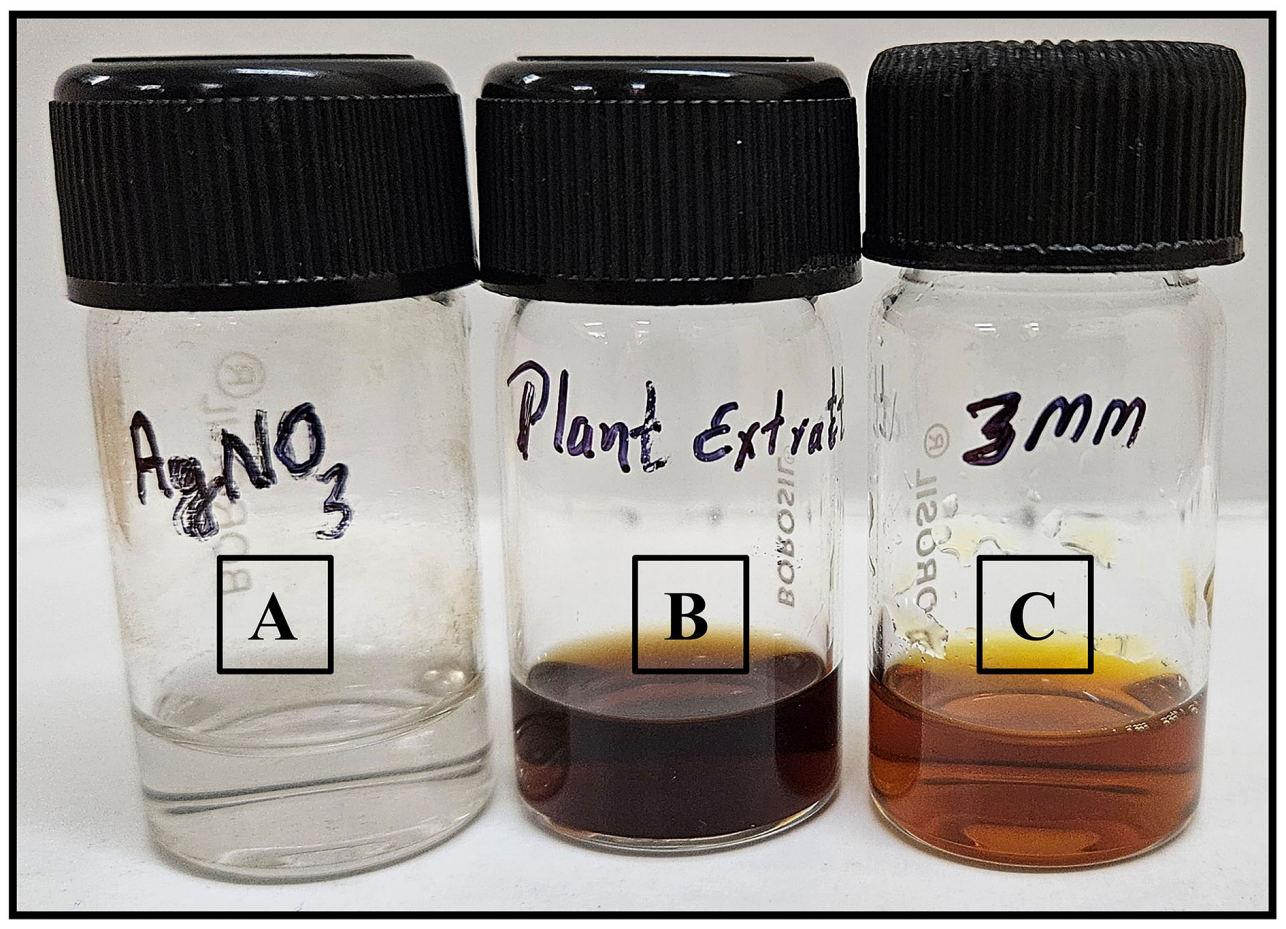
**(A)** Solution of silver nitrate (AgNO_3_), **(B)** the plant extract, and **(C)** synthesized 3 mM AgNPs.

**Scheme 1:**
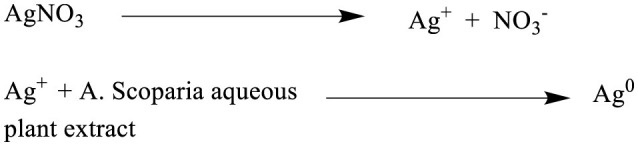
Schematic representation of the synthesis of AgNPs (Ag°) using *A. scoparia* aqueous plant extract. As shown in Scheme 1, the process involves the dissociation of AgNO_3_ into silver ions (Ag^+^) and nitrate ions (${\text{NO}}_{3}^{-}$), followed by the reduction of Ag^+^ to elemental silver (Ag°) through the reducing and stabilizing action of phytochemicals present in the plant extract, culminating in the formation of AgNPs.

To synthesize AgNPs through a green method, 5 mL of fresh plant extract was slowly added to 95 mL of a 3 mM AgNO_3_ solution in an Erlenmeyer flask. The mixture was gently heated to 45°C and continuously stirred. The formation of nanoparticles was indicated by a color change from pale yellowish to reddish brown (Figure 2, Scheme 1). After allowing the reaction to proceed for 24 h or until reaching room temperature, the mixture was centrifuged at 5,000 rpm for 30 min to isolate the nanoparticles. The supernatant was carefully discarded, and the nanoparticles were dried at 25°C before being stored in labeled vials. UV-vis spectroscopy was used to confirm the successful synthesis of the AgNPs (Fahim et al., 2024).

## 3 Characterizations of synthesized silver nanoparticles (AgNPs)

### 3.1 UV–visible spectrum analysis (UV-VIS)

The formation of AgNPs through the bioreduction of silver ions (Ag^+^) was confirmed by UV-visible spectrophotometry Thermo Scientific (Multiskan Go) with a 1 nm resolution over a wavelength range of 200–800 nm. Spectral measurements were taken at room temperature at various intervals, using distilled water as a baseline reference, and a 2 mL sample pipetted into the cuvette. The analysis of absorption peaks in this range verified the presence and stability of AgNPs in the colloidal solution (Labulo et al., 2022).

### 3.2 Fourier transform infrared analysis (FTIR)

The purified AgNPs analysis was conducted using Fourier Transform Infrared (FTIR) spectroscopy in ATR mode to determine the contributions of phytochemicals from the plant extracts in modifying their surfaces. Using a PerkinElmer Spectrum FTIR spectrometer at room temperature, the spectra were recorded over a 4,000–400 cm^−1^ wavelength range. This investigation identified significant interactions between silver and bioactive molecules, as evidenced by distinct peaks that indicate the formation and stabilization of AgNPs. The FTIR results for AgNPs were compared to those of the plant extract to highlight the key biomolecules in reducing silver ions (Ghojavand et al., 2020).

### 3.3 Transmission electron microscopic analysis (TEM)

The AgNPs' morphological characteristics and size distribution were examined using TEM analysis with a Tecnai G2 20 S-TWIN [FEI] at 200 kV. Sample preparation involved dilution, sonication, filtration, and placing a drop onto carbon-coated copper grids for air-drying (Sreelekha et al., 2021).

### 3.4 Field emission scanning electron microscopy analysis (FESEM)

The AgNPs' surface morphology, shape, size, crystalline structure, and elemental composition were analyzed using FESEM on TESCAN MIRA-LMS. Samples were prepared as thin films on carbon-coated copper grids, dried, and examined confirming the silver presence and other elemental compositions (Labulo et al., 2022).

### 3.5 DLS zeta analysis (particle analyzer)

Dynamic light scattering (DLS) and zeta potential analysis on an Anton Paar Litesizer 500 were used to determine the synthesized AgNPs' size distribution, surface charge, and stability, providing a detailed profile of their suspension stability (Mahiuddin et al., 2020).

## 4 Biological applications and chemical applications

### 4.1 Antibacterial activity

The antibacterial activity of AgNPs was evaluated using the agar disc diffusion method against two bacterial strains: *Enterococcus faecalis (E. faecalis)* (Ali et al., 2021) and *Pseudomonas aeruginosa (P. aeruginosa)* (Muddassir et al., 2022), obtained from the Amity Institute of Biotechnology at Amity University Rajasthan, Jaipur. Bacterial cultures were grown in Luria-Bertani Broth (LBB) for 12 h at 37°C in an orbital shaker set at 180 rpm to ensure proper aeration (Kim et al., 2020). For testing, fresh Luria-Bertani Agar (LBA) plates were prepared, and 6 mm sterile filter paper discs were impregnated with 3 mM AgNPs and the corresponding plant extract. The discs were then placed on the surface of agar plates inoculated with the bacterial strains. Ciprofloxacin (CIP) and Kanamycin (KAN) were used as positive controls, while distilled water was the negative control. The plates were incubated for 24 h at 37°C, allowing bacterial growth (Hidayat et al., 2023). After incubation, the zones of inhibition around the discs were measured in millimeters to assess the antibacterial effectiveness of the AgNPs. The antimicrobial efficacy of the AgNPs was further quantified by determining the minimum inhibitory concentration (MIC), where applicable.

### 4.2 Catalytic reduction of nitro compounds using synthesized AgNPs

The catalytic reduction activity of AgNPs synthesized using *A. scoparia* extract was evaluated on four nitroaromatic compounds: 1-bromo-4-nitrobenzene, 4-bromo-2-fluoro-1-nitrobenzene, 4-nitroaniline, and 4-nitrophenol (Singh et al., 2018). Each nitro compound was subjected to a reduction reaction with AgNPs as a catalyst. Sodium borohydride (NaBH4), served as the reducing agent, facilitating electron transfer and hydrogenation of the nitro groups to their respective amine forms (Gondwal et al., 2023). The reaction was monitored via UV-vis spectroscopy, tracking the decrease in absorbance at the characteristic wavelengths of the nitro compounds as they were converted into their reduced products. This reduction was indicated by the progressive disappearance of the nitro peak and the emergence of a new peak corresponding to the amine product.

## 5 Result and discussion

### 5.1 Bioactive compounds in the plant extract confirmatory tests

Table 1 shows the phytochemical composition of the plant extract. The aqueous extract of *A. scoparia* confirmed the presence of phenols, tannins, flavonoids, Saponins, and Carbohydrates.

**Table 1:** Bioactive compounds in the plant extract of *A.scoparia*.

**S. no**.	**Test for bioactive compounds**	**Test name**	**Observation**	**Result**
1.	Phenols and tannins	Ferric chloride test	A color change to blue, green, or purple indicates the presence of phenols and tannins.	Present
2.	Flavonoids	Alkaline test	The yellow color upon adding NaOH indicates the presence of flavonoids. The yellow color disappears or turns colorless upon adding dilute acid, confirming flavonoids.	Present
3.	Saponins	Foam test	The formation of stable foam that persists for 10–15 min after shaking indicates the presence of saponins.	Present
4.	Carbohydrates	Molisch test	A violet or reddish-brown ring at the interface between the acid and extract layers indicates the presence of carbohydrates.	Present

### 5.2 Characterizations of silver nanoparticles (AgNPs)

In this section, the structural, morphological, and compositional properties of AgNPs synthesized using *A. scoparia* extract were characterized using various advanced techniques. The results from each analytical method are detailed as follows.

#### 5.2.1 UV–vis spectrum

**Figure 3:**
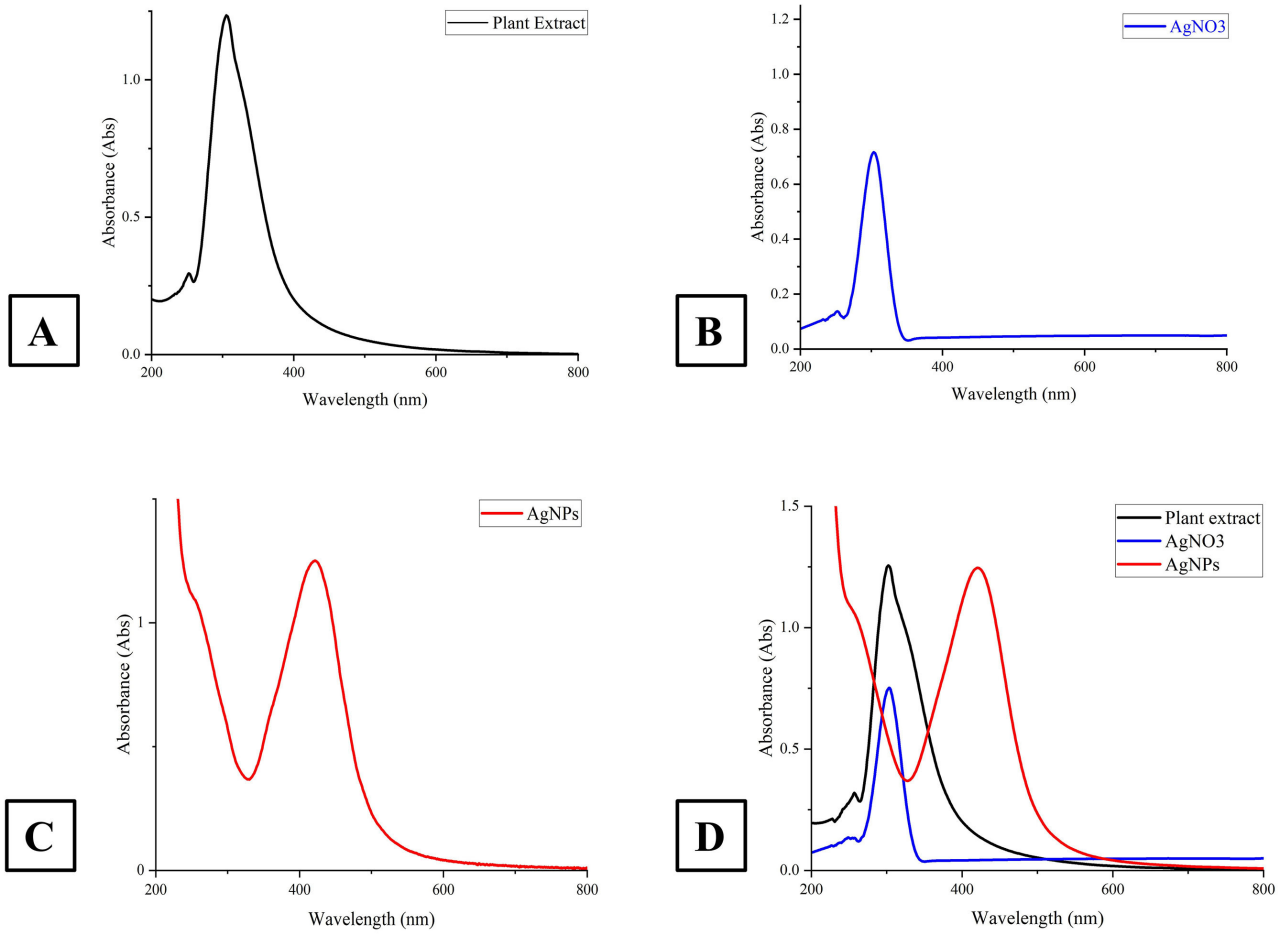
This figure presents the UV-Vis absorption spectra of *A. scoparia* plant extract, silver nitrate (AgNO_3_) solution, and AgNPs. **(A)** spectrum of the plant extract; **(B)** shows the spectrum of the AgNO_3_ solution; **(C)** depicts the absorption spectrum of the synthesized AgNPs at 421 nm, indicating successful nanoparticle formation and **(D)** compares the spectra of the plant extract (black), AgNO_3_ solution (blue), and AgNPs (green), emphasizing the shift in the absorption peak as the synthesis progresses.

The UV–vis spectra of AgNO_3_, the plant extract, the synthesized AgNPs and colloidal solutions are presented in Figure 3. The sharp absorption bands confirm the characteristic peaks at 303 nm for AgNO_3_, 290 nm for the plant extract, and 421 nm for the AgNPs, respectively, corroborating successful nanoparticle synthesis.

#### 5.2.2 FTIR

**Figure 4:**
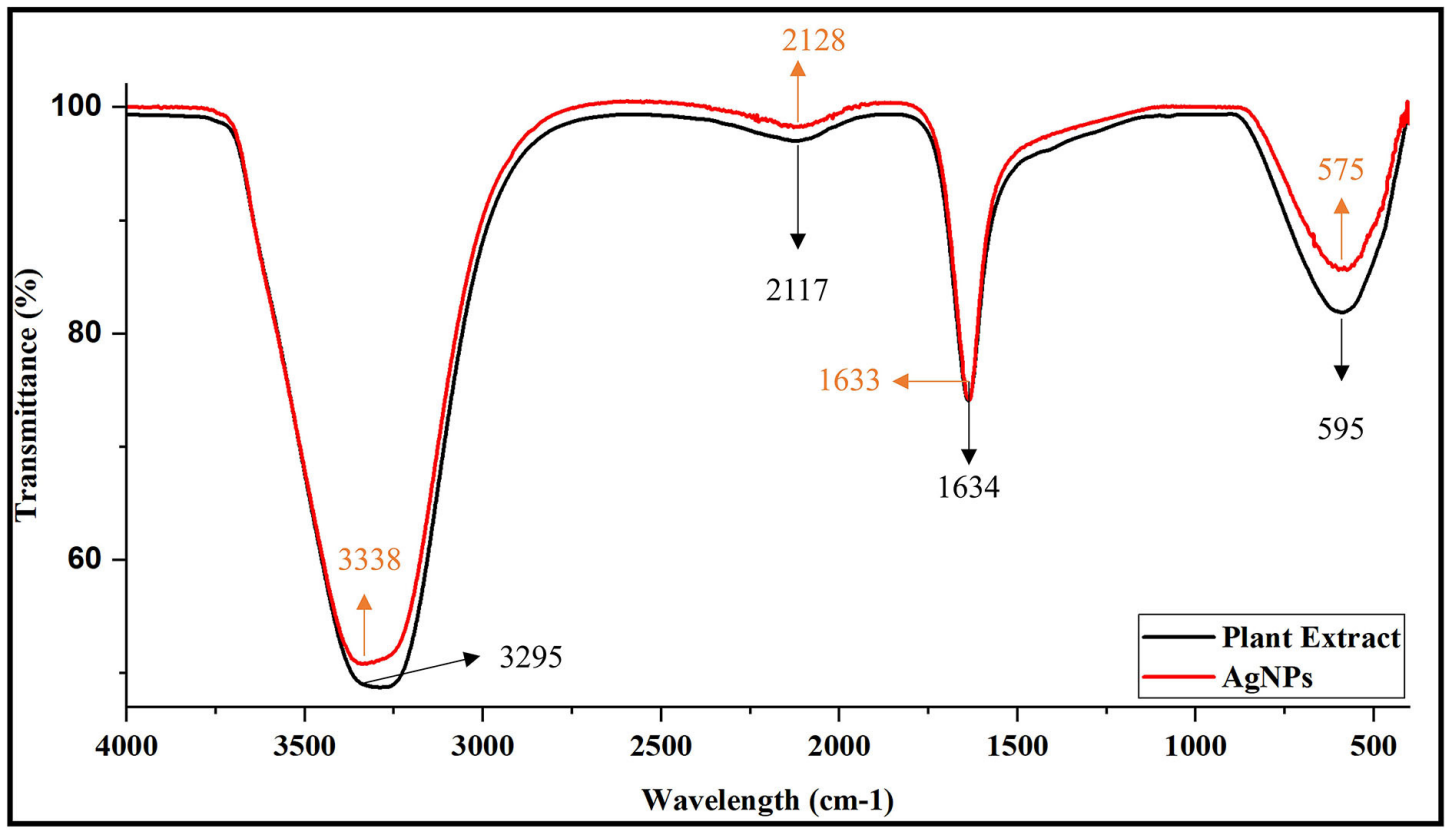
FTIR spectra of plant extract and synthesized AgNPs.

The FTIR spectra of the plant extract and the synthesized AgNPs reveal important functional groups and their interactions during nanoparticle synthesis. In the plant extract, prominent peaks are observed at 3,295 cm^−1^ (hydroxyl, –OH group), 2,117 cm^−1^ (nitrile, C=N group), 1,634 cm^−1^ (amide carbonyl, C=O group), and 595 cm^−1^ (likely aromatic C–H out-of-plane bending). The FTIR spectrum of the synthesized AgNPs shows slight shifts in these peaks: the hydroxyl group peak moves to 3,338 cm^−1^, the nitrile group peak shifts to 2,128 cm^−1^, and the amide carbonyl peak appears at 1,633 cm^−1^, suggesting interactions with the AgNPs. Additionally, the peak at 595 cm^−1^ in the plant extract shifts to 575 cm^−1^ in the AgNPs, likely due to the formation of silver-oxygen (Ag–O) bonds. These spectral changes confirm that the bioactive compounds in the plant extract play a crucial role in both reducing and stabilizing the AgNPs (Figure 4).

#### 5.2.3 TEM

**Figure 5:**
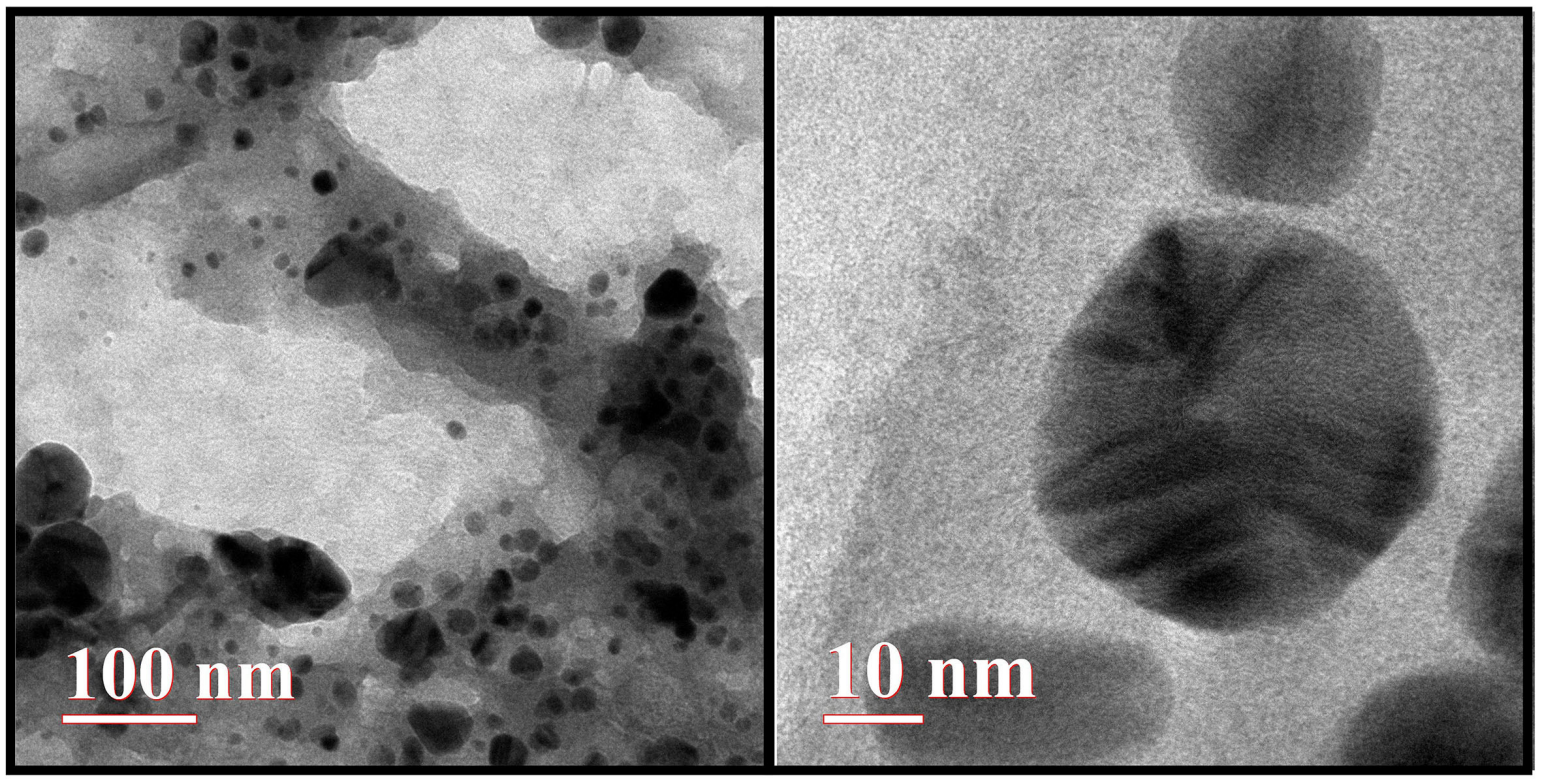
Transmission electron microscopy (TEM) image of AgNPs.

As shown in Figure 5, the transmission electron microscopy (TEM) analysis revealed that the synthesized AgNPs were predominantly spherical with a uniform morphology. Minimal agglomeration was observed, and the TEM images highlight the successful synthesis and stability of the AgNPs.

#### 5.2.4 FESEM

**Figure 6:**
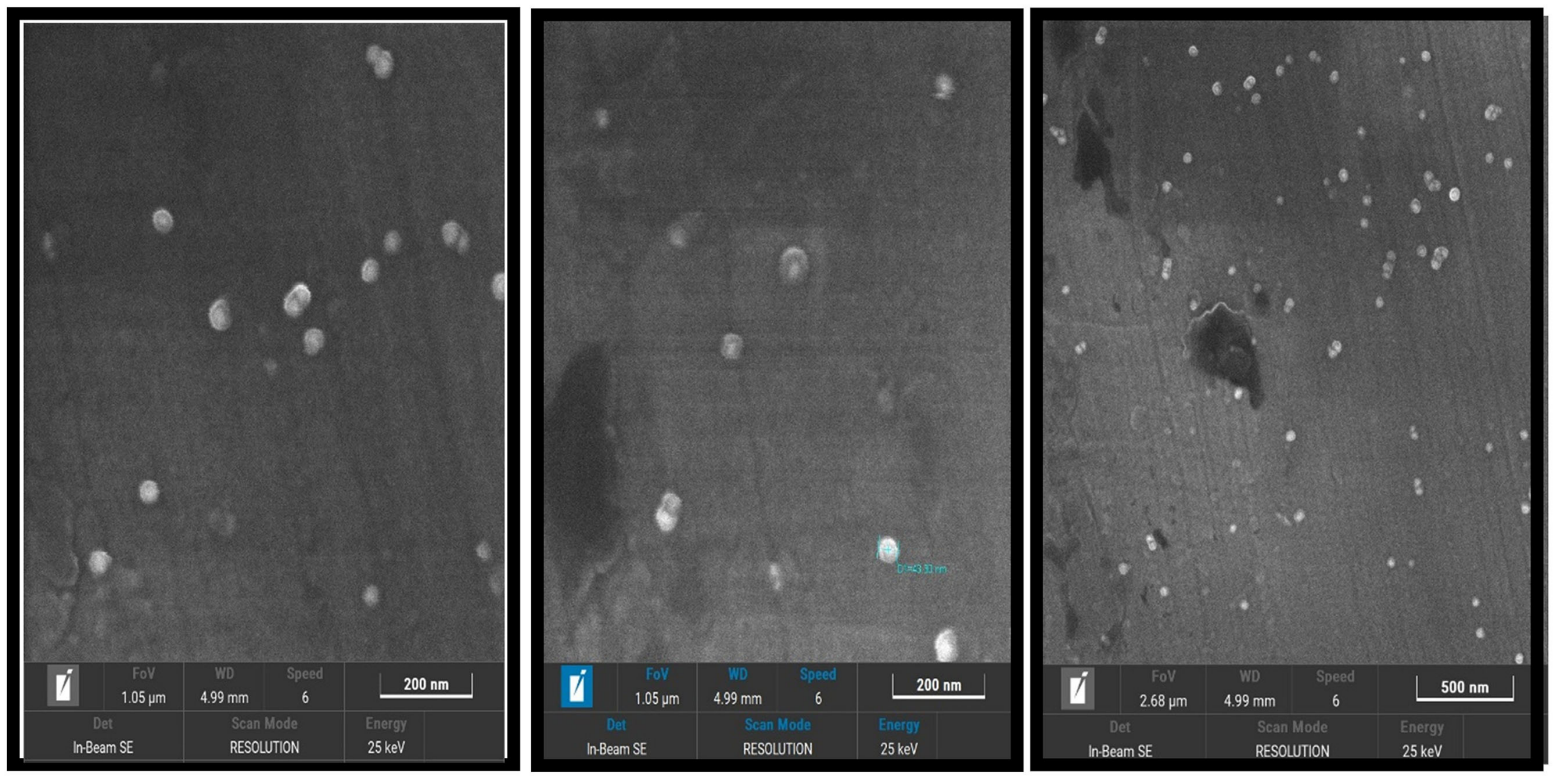
Field emission scanning electron microscopy (FESEM) image of AgNPs.

The field emission scanning electron microscopy (FESEM) analysis confirmed the formation of AgNPs with predominantly spherical shapes and smooth surfaces. The nanoparticles were uniformly distributed, with an average size of ~43.30 nm. Minimal agglomeration observed in the FESEM images indicates the stability of the synthesized AgNPs, further validating the success of the synthesis method (Figure 6).

#### 5.2.5 DLS zeta

The dynamic light scattering (DLS) and zeta potential analysis provided valuable insights into the size distribution and surface charge of the synthesized AgNPs. The DLS results confirmed a uniform size distribution with an average hydrodynamic diameter of ~232.9 nm, indicating the formation of nanoparticles within the desired range. The relatively narrow polydispersity index (PDI) value of 0.148 demonstrated the homogeneity of the synthesized AgNPs.

**Figure 7:**
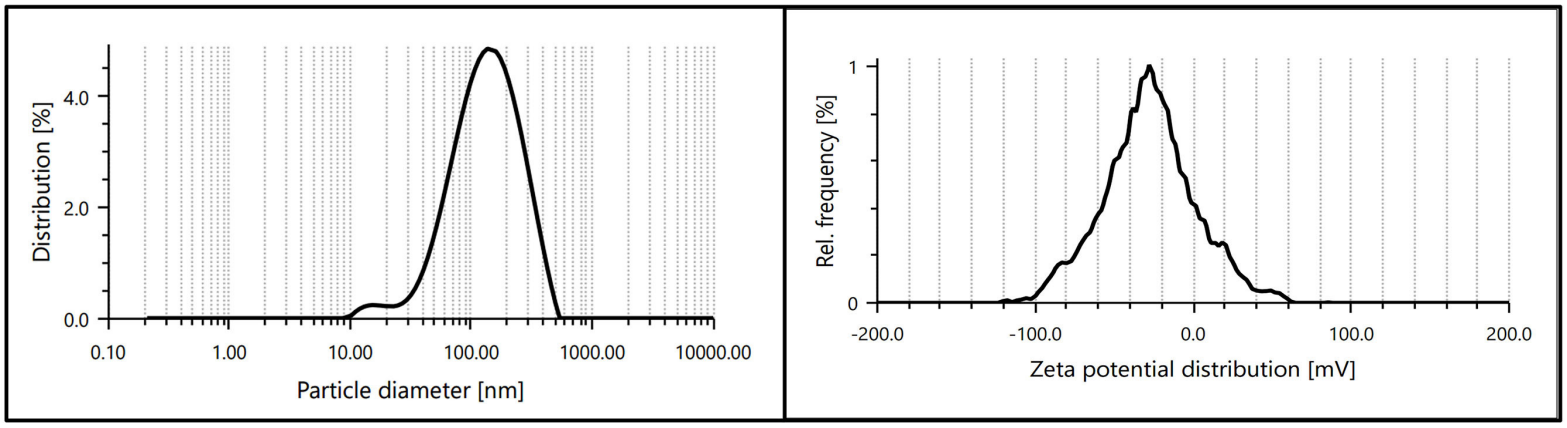
Particle size distribution and zeta potential of synthesized AgNPs, indicating uniform size and high colloidal stability.

The zeta potential measurements revealed a surface charge of −25.3 mV, indicating significant electrostatic stability of the nanoparticles in the colloidal solution. The high zeta potential value suggests strong repulsive forces between particles, preventing aggregation and ensuring dispersion stability. These findings collectively confirm the successful synthesis of stable and uniformly dispersed AgNPs (Figure 7).

### 5.3 Biological applications and chemical applications

#### 5.3.1 Antibacterial activity

**Figure 8:**
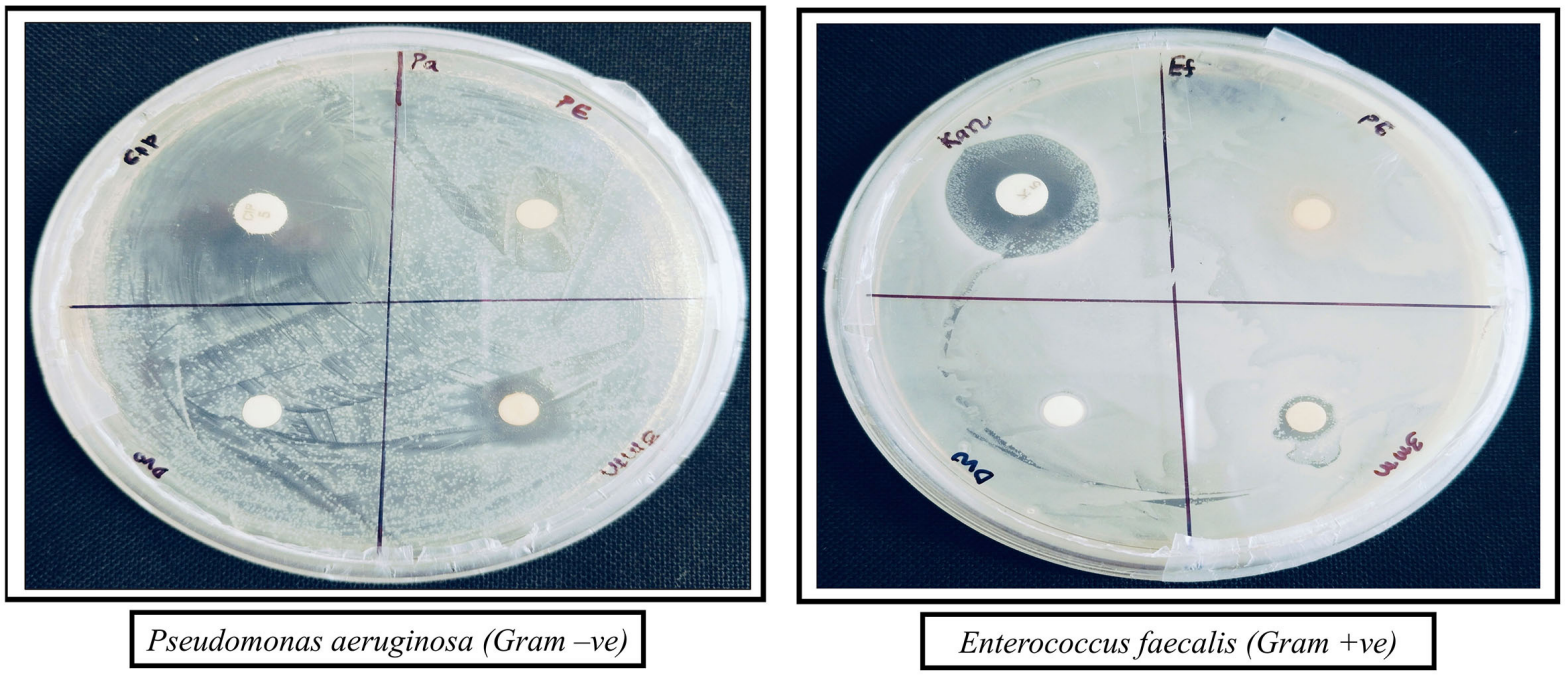
Antibacterial activity of AgNPs against *P. aeruginosa* (Gram-negative) and *E. faecalis* (Gram-positive). The zones of inhibition show the effectiveness of different treatments: Antibiotic (positive control), DW (negative control), PE (plant extract), and AgNPs (silver nanoparticles).

**Figure 9:**
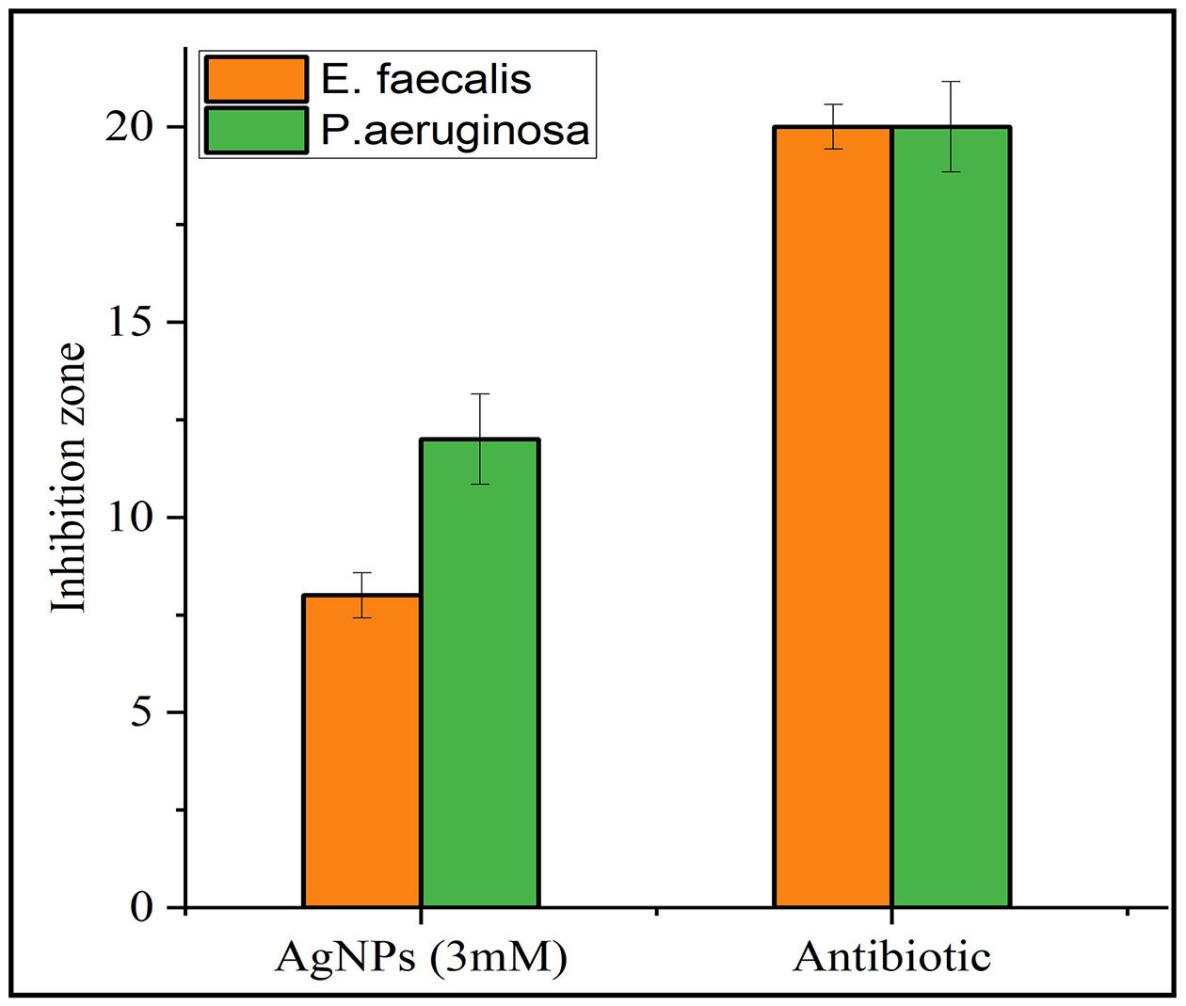
Comparative inhibition zones (in mm) for *E. faecalis* and *P. aeruginosa* treated with AgNPs and a standard antibiotic. Orange bars represent the inhibition zones for *E. faecalis*, while green bars represent those for *P. aeruginosa*. The results demonstrate antimicrobial activity for both AgNPs and the antibiotic against the tested strains.

The antibacterial activity of the synthesized AgNPs was evaluated against *P. aeruginosa* (Gram-negative) and *E. faecalis* (Gram-positive) using the agar disc diffusion method. The positive control (antibiotic) showed the largest inhibition zones, while the negative control (distilled water) displayed no antibacterial activity (Figure 8). AgNPs (3 mM) demonstrated strong antibacterial effects, with inhibition zones of 8 ± 0.577 mm for *E. faecalis* and 12 ± 1.155 mm for *P. aeruginosa*, which were larger than those observed with the plant extract alone. All experiments were conducted in triplicate to ensure reliability, and the results are presented as mean ± standard error (SE) (Figure 9).

The SE was calculated using the formula:


$$\begin{array}{l} SE=\frac{SD}{\sqrt{n}} \end{array}$$

where:

SD: represents the standard deviation.

*n*: represents the number of replicates.

These results demonstrate that biosynthesized AgNPs are effective antimicrobial agents, likely due to their small size and large surface area.

#### 5.3.2 Catalytic reduction of nitro compounds using synthesized AgNPs

**Figure 10:**
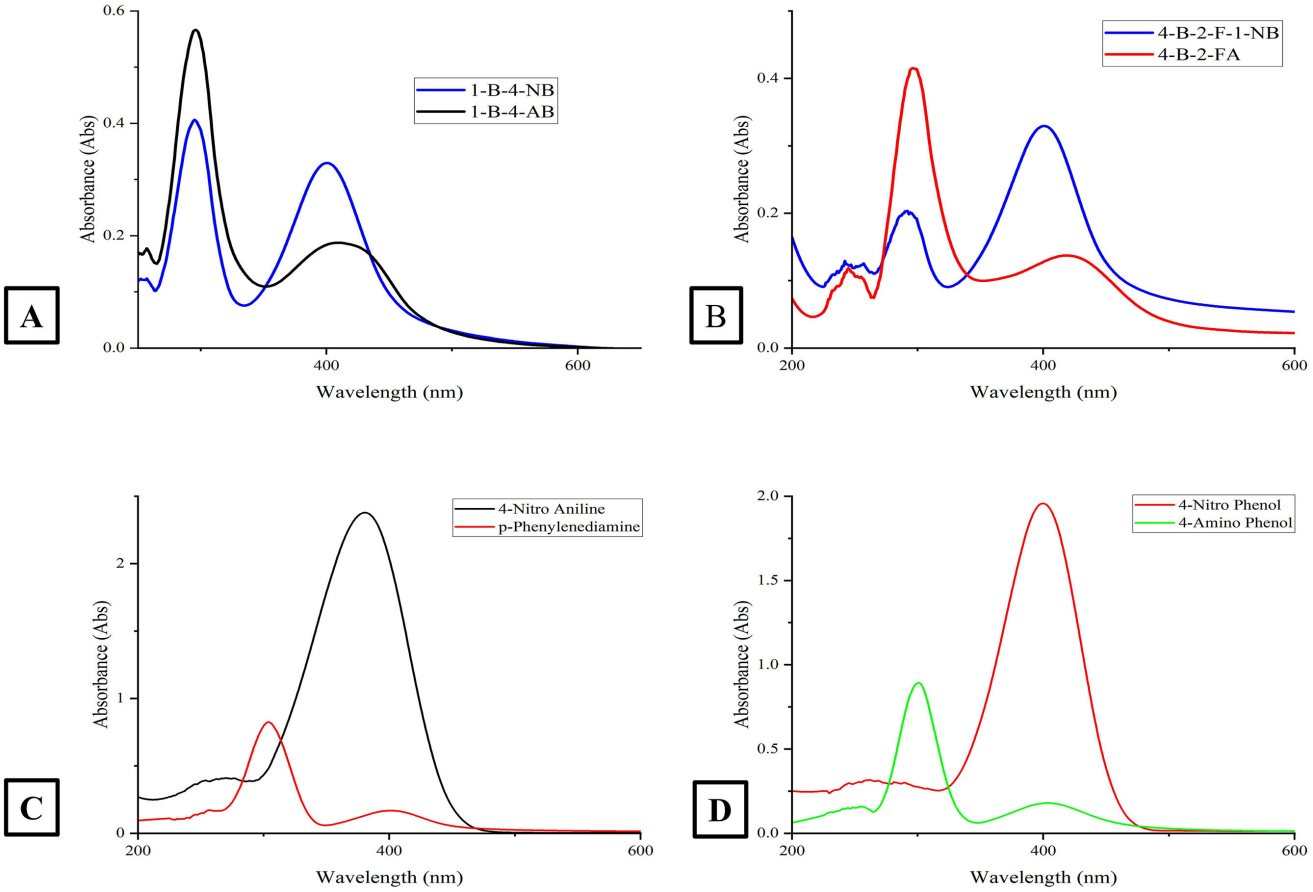
This figure presents four absorption spectra plots (labeled **A–D**) for various chemical compounds. **(A)** Displays the absorption spectra for the compounds 1-Br-4-NB and 1-Br-4-AB; **(B)** shows the absorption spectra for the compounds 4-Br-2-F-1-NB and 4-Br-2-F-1-A; **(C)** presents the absorption spectra for 4-Nitro Aniline and p-phenylenediamine and **(D)** depicts the absorption spectra for 4-Nitro Phenol and 4-Amino Phenol. The *x*-axis in all subplots represents the wavelength in nanometers (nm), and the *y*-axis shows the absorbance. Each plot identifies the specific compounds being displayed.

**Scheme 2:**
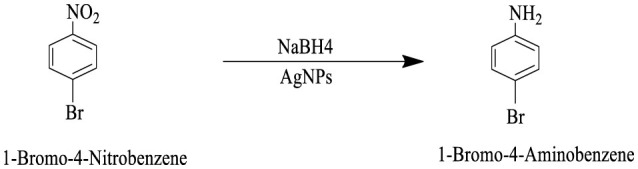
This reaction scheme depicts the reduction of 1-bromo-4-nitrobenzene to 1-bromo-4-aminobenzene using sodium borohydride (NaBH4), and AgNPs as reducing agents. The nitro group (NO2), on the starting material is converted to an amino group (NH2), in the product.

**Scheme 3:**
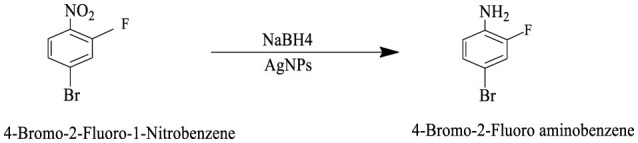
This reaction scheme shows the reduction of 4-bromo-2-fluoro-1-nitrobenzene to 4-bromo-2-fluoroaniline using sodium borohydride (NaBH4), and AgNPs as reducing agents. The nitro group (NO2), is converted to an amino group (NH2), in the product.

**Scheme 4:**
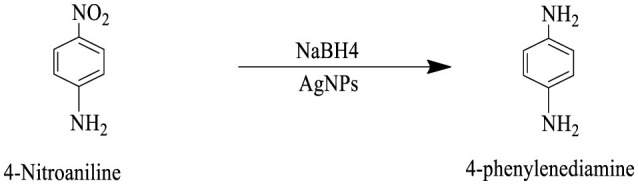
This reaction scheme represents the reduction of 4-nitroaniline to 4-phenylenediamine using sodium borohydride (NaBH4), and AgNPs as reducing agents. The nitro group (NO2), is converted to an amino group (NH2), in the final product.

**Scheme 5:**
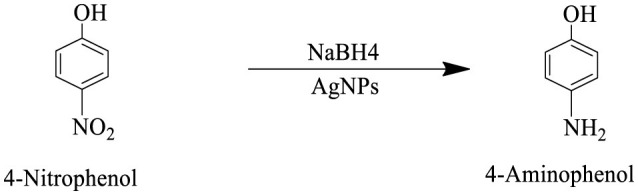
The reaction scheme represents the chemical reduction of 4-nitrophenol to 4-aminophenol, utilizing sodium borohydride (NaBH4), and AgNPs. The nitro group (NO2), is successfully reduced to an amino group (NH2).

**Figure 11:**
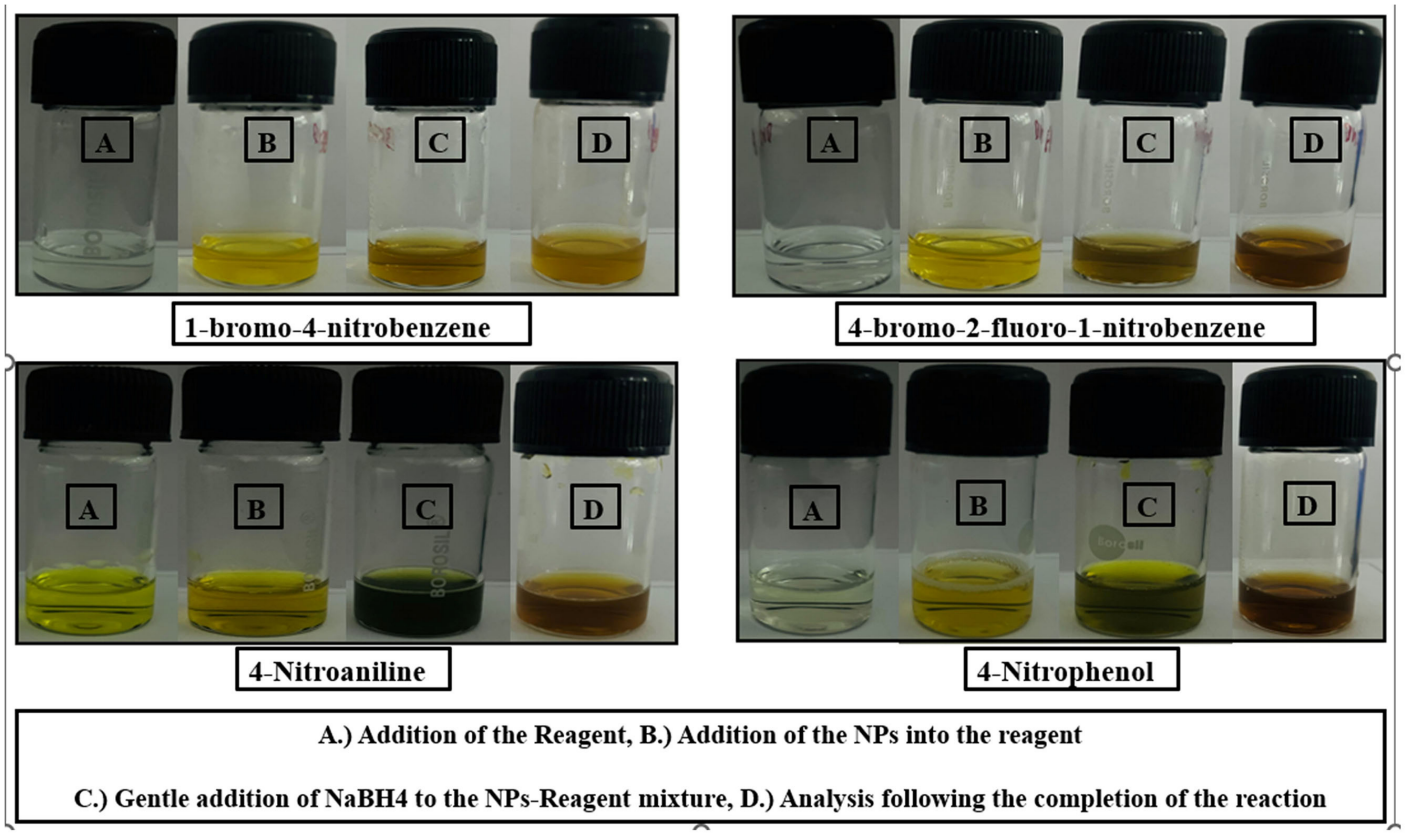
This figure presents a series of images showcasing the visual changes observed during the reaction processes for four different chemical compounds: 1-bromo-4-nitrobenzene, 4-bromo-2-fluoro-1-nitrobenzene, 4-nitroaniline, and 4-nitrophenol. **(A)** Addition of the reagent, **(B)** Addition of the NPs into the reagent, **(C)** Gentle addition of NaBH4 to the Nps-reagent mixture, **(D)** Analysis following the completion of the reaction.

The catalytic potential of the synthesized AgNPs was comprehensively demonstrated through the reduction of various nitroaromatic compounds using NaBH4, monitored via UV-vis spectroscopy. The reduction of 1-bromo-4-nitrobenzene (1-Br-4-NB) to 4-bromoaniline (4-BrA) was evidenced by the reduction of the 365 nm peak and the emergence of a new peak at 300 nm, achieving a 44.22% reduction within 20 min and accompanied by a visible color change (Figure 10A, Scheme 2). Similarly, the reduction of 4-bromo-2-fluoro-1-nitrobenzene (4-Br-2-F-1-NB) to 4-bromo-2-fluoroaniline (4-Br-2-F-An) showed a decline of the 401 nm peak and the appearance of a 288 nm peak, with a 61.20% reduction in 30 min (Figure 10B, Scheme 3). The reduction of 4-nitroaniline (4-NA) to 4-phenylenediamine (4-PDA) was marked by the disappearance of the 381 nm peak and the emergence of a 305 nm peak, achieving a 92.91% reduction within 25 min, accompanied by a color change (Figure 10C, Scheme 4). Finally, the reduction of 4-nitrophenol (4-NP) to 4-aminophenol (4-AP) was characterized by an initial shift of the 316 nm peak to 400 nm upon the addition of NaBH4, followed by a reduction to 299 nm, achieving a 90.90% reduction within 15 min, along with a yellowish-green color change indicating the formation of 4-nitrophenolate ions (Figure 10D, Scheme 5). In all cases, negligible changes were observed in the absence of AgNPs, underscoring the kinetic barriers of the uncatalyzed reactions and highlighting the remarkable catalytic efficiency of AgNPs in facilitating these transformations (Figure 11).

To calculate the Efficiency (%), rate constant **(*k*)** and half-life (*t*_1/2_), we use the following first-order kinetic equations:

**Efficiency** (**%**)**:**


$$\begin{array}{l} \frac{0}{0}=\left(\frac{{\text{a}}_{0}-{\text{a}}_{\text{t}}}{{\text{a}}_{0}}\right)\times 100 \end{array}$$

where:

a_0_: Initial absorbance at the characteristic wavelength of the nitro compound.

a_t_: Absorbance at the same wavelength after a specific time.

**Rate constant *k*:**


$$\begin{array}{l} \ln\frac{\left[A_{t}\right]}{\left[A_{0}\right]}=-kt \end{array}$$

where:

*A*_0_: The initial absorbance of the peak.

*A*_*t*_: The final absorbance of the peak after time *t* (reaction time).

*k*: The rate constant for the reaction, specific to the reaction conditions such as temperature.

*t*: The time elapsed since the start of the reaction.

**Half-life *t***_1/2_:


$$\begin{array}{l} t_{\frac{1}{2}}=\frac{0.693}{k} \end{array}$$

where:

0.693 (ln), a constant.

*k*: The first-order rate constant (in min^−1^).

Based on the calculated rate constants **(*k*)** for the reduction of the nitroaromatic compounds, the rate of reduction follows the order:

4-Nitrophenol (4-NP) > 4-Nitroaniline (4-NA) > 4-Bromo-2-fluoro-1-nitrobenzene (4-Br-2-F-1-NB) > 1-Bromo-4-nitrobenzene (1-Br-4-NB) shows in Table 2 (Gondwal et al., 2023).

**Table 2:** Shows kinetic parameters for the reduction of nitroaromatic compounds catalyzed by AgNPs.

**S. no**.	**Nitro compounds**	**Calculated rate constant *k* (min^−^1)**	**Half-life *t*_1/2_ (min)**	**Efficiency (%)**
1	1-Bromo-4-nitrobenzene (1-Br-4-NB)	0.016	43.43	44.22
2	4-Bromo-2-fluoro-1-nitrobenzene (4-Br-2-F-1-NB)	0.0316	21.97	61.2
3	4-Nitroaniline (4-NA)	0.1148	6.04	92.91
4	4-Nitrophenol (4-NP)	0.1597	4.34	90.9

The figure is divided into four rows, with each row representing a different compound. Within each row, there are four labeled vials (A, B, C, D) depicting the visual changes at various stages of the reaction, as described in the caption:

**(A)** Addition of the Reagent.

**(B)** Addition of the NPs into the reagent.

**(C)** Gentle addition of NaBH4 to the NPs-Reagent mixture.

**(D)** Analysis following the completion of the reaction.

The images provide a clear visual representation of the reaction processes and the resulting changes in the sample appearance for the four compounds under investigation.

## 6 Conclusion

This study demonstrates the successful green synthesis of silver nanoparticles (AgNPs) using *Artemisia scoparia (A. scoparia)* extract, offering an eco-friendly and sustainable approach to nanoparticle production. Phytochemical screening of *A. scoparia* revealed the presence of phenols, tannins, flavonoids, saponins, and carbohydrates, which acted as natural reducing and capping agents during synthesis. Comprehensive characterization through UV-vis spectroscopy, FTIR, FE-SEM, TEM, and DLS Zeta analysis confirmed the stable morphology of the AgNPs. The synthesized AgNPs exhibited significant antibacterial activity against both Gram-positive (*E. faecalis*) and Gram-negative (*P. aeruginosa*) bacteria, highlighting their potential as effective antimicrobial agents. Additionally, their catalytic efficiency in the reduction of nitro compounds demonstrated their suitability for industrial and environmental applications. Overall, the use of *A. scoparia*-derived phytochemicals for AgNP synthesis not only reduces environmental impact but also enhances the nanoparticles' functional properties, opening avenues for their application in biomedical, catalytic, and environmental fields. These findings pave the way for future research focused on expanding their applications in drug delivery, advanced catalysis, and sustainable environmental solutions.

## Data Availability

The original contributions presented in the study are included in the article/supplementary material, further inquiries can be directed to the corresponding author/s.
